# Preliminary Findings of the High Quantity of Microplastics in Faeces of Hong Kong Residents

**DOI:** 10.3390/toxics10080414

**Published:** 2022-07-23

**Authors:** Yuen-Wa Ho, Jin Yan Lim, Yun Kit Yeoh, Jia-Chi Chiou, Yuyan Zhu, Keng Po Lai, Lei Li, Paul Kay Sheung Chan, James Kar-Hei Fang

**Affiliations:** 1 Department of Applied Biology and Chemical Technology, The Hong Kong Polytechnic University, Hung Hom, Hong Kong 999077, China; yuenwa.ho@polyu.edu.hk (Y.-W.H.); jiachi.amber.chiou@polyu.edu.hk (J.-C.C.); yuyan.zhu@polyu.edu.hk (Y.Z.); 2 Department of Microbiology, The Chinese University of Hong Kong, Prince of Wales Hospital, Sha Tin, Hong Kong 999077, China; jylim@cuhk.edu.hk (J.Y.L.); yeohyunkit@cuhk.edu.hk (Y.K.Y.); paulkschan@cuhk.edu.hk (P.K.S.C.); 3 Centre for Gut Microbiota Research, The Chinese University of Hong Kong, Prince of Wales Hospital, Sha Tin, Hong Kong 999077, China; 4 Microbiota I-Centre, Sha Tin, Hong Kong 999077, China; 5 Research Institute for Future Food, The Hong Kong Polytechnic University, Hung Hom, Hong Kong 999077, China; 6 Guangxi Key Laboratory of Tumor Immunology and Microenvironmental Regulation, Guilin Medical University, Guilin 541004, China; kengplai@cityu.edu.hk; 7 Department of Chemistry, City University of Hong Kong, Kowloon Tong, Hong Kong 999077, China; 8 Shenzhen Institute of Advanced Technology, Chinese Academy of Sciences, Shenzhen 518055, China; saralilei@siat.ac.cn; 9 State Key Laboratory of Marine Pollution, City University of Hong Kong, Kowloon Tong, Hong Kong 999077, China

**Keywords:** microplastic, stool, gastrointestinal tract, gut microbiota, South China

## Abstract

Microplastics are recognised as a ubiquitous and hazardous pollutant worldwide. These small-sized particles have been detected in human faeces collected from a number of cities, providing evidence of human ingestion of microplastics and their presence in the gastrointestinal tract. Here, using Raman spectroscopy, we identified an average of 50 particles g^−1^ (20.4–138.9 particles g^−1^ wet weight) in faeces collected from a healthy cohort in Hong Kong. This quantity was about five times higher than the values reported in other places in Asia and Europe. Polystyrene was the most abundant polymer type found in the faeces, followed by polypropylene and polyethylene. These particles were primarily fragments, but about two-thirds of the detected polyethylene terephthalate were fibres. More than 88% of the microplastics were smaller than 300 µm in size. Our study provides the first data on the faecal level, and thus the extent of ingestion, of microplastics in Hong Kong’s population. This timely assessment is crucial and supports the recently estimated ingestion rate of microplastics by Hong Kong residents through seafood consumption, which is one of the highest worldwide. These findings may be applicable to other coastal populations in South China with similar eating habits.

## 1. Introduction

Alongside the more widespread use of plastics that can be manufactured at a cheaper cost but with higher durability, the magnitude of plastic pollution has increased drastically worldwide over the past two decades [1]. For instance, it has been estimated that up to 2.4 million tonnes of plastic waste is annually discharged into the ocean through various human activities [2]. These plastic pieces can be fragmented into smaller sizes, and when smaller than 5 mm, they are commonly referred to as microplastics, which can be found nowadays almost everywhere and in a wide range of food items such as fish, shellfish and table salt, as well as drinking water (e.g., [3,4,5]). Microplastics have also been detected in human faeces collected from different populations in Asia, Europe and America, suggesting human ingestion of microplastics and their presence in the gastrointestinal tract [6,7,8,9,10]. However, details on human exposure to microplastics are still far from sufficient. Here, we supplement this information by determining the microplastic content in faeces of a healthy cohort in Hong Kong and assessing the exposure relevant to the local setting [11]. This assessment is timely, since the ingestion rate of microplastics by Hong Kong residents through seafood consumption has been recently estimated to be one of the highest worldwide [5].

## 2. Materials and Methods

Microplastics were extracted from eight faecal samples collected from four men and four women aged 30–65 as part of an established gut microbiota survey of the Hong Kong population in September 2020 [12]. Each faecal sample was collected with a wooden stick and stored at −20 °C prior to laboratory processing. The frozen sample was thawed and an aliquot (0.32 ± 0.14 g; mean ± SD) was digested in a 450 mL solution of 10% potassium hydroxide (KOH; Acros Organics, Geel, Belgium) and 15% ethylenediaminetetraacetic acid disodium salt dihydrate (EDTA; Acros Organics) at 40 °C for 24 h, after which 50 mL of 30% hydrogen peroxide (H_2_O_2_; Sigma Aldrich, St. Louis, MO, USA) was added and the digestion process continued for another 48 h [13]. Any undigested matter was collected by filtration and further digested in 50 mL of 2% 1-allyl-3-methylimidazolium chloride (AMIM-Cl; Sigma Aldrich) for 24 h to remove cellulose fibres [14]. All particles were retrieved by filtration and resuspended in a 50 mL dense solution of sodium iodide (3.67 g cm^−3^; Sigma Aldrich) to separate microplastics by flotation. Microplastics in the supernatant were retrieved on a stainless-steel sieve with 30 µm pores. To minimise contamination, all solutions were prepared using ultrapure water prefiltered through 0.22 µm pores (Merck Milli-Q, Darmstadt, Germany). All glassware and tools were thoroughly rinsed with ultrapure water before use, and cotton lab coats and nitrile gloves were worn at all times during sample processing. Eight procedural blank samples using ultrapure water were included to estimate the background level of microplastics.

**Figure 1:**
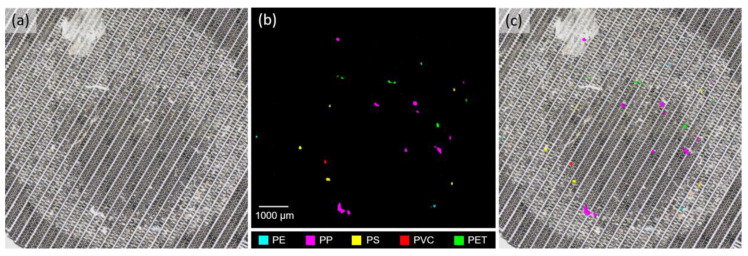
(**a**) Microplastics and other undigested matter extracted from human faeces on a stainless-steel sieve with 30 μm pores, (**b**) a two-dimensional array of colour-coded microplastics generated with an automated Raman mapping approach at a spatial resolution of 28.4 µm, and (**c**) the superimposed image of (**a**) and (**b**) to locate these particles, which were confirmed to be polyethylene (PE), polypropylene (PP), polystyrene (PS), polyvinyl chloride (PVC) and polyethylene terephthalate (PET).

**Figure 2:**
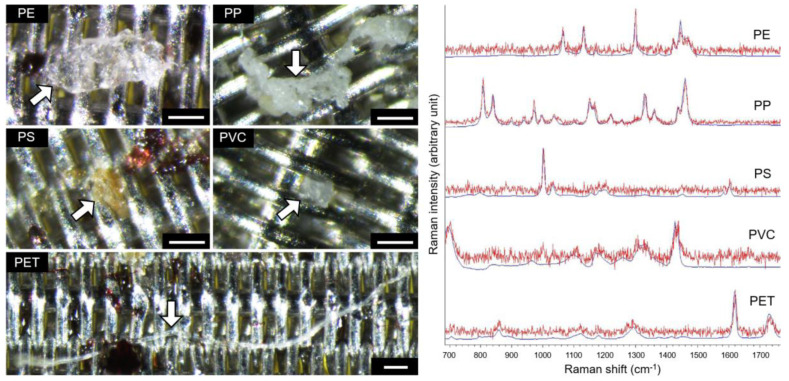
Selected microplastics on stainless-steel sieves with a plain Dutch weave pattern (scale bar: 100 µm), and their Raman spectra (red) compared to the reference spectra of PE, PP, PS, PVC and PET provided in the Renishaw Polymeric Materials Database (blue). Please refer to Figure 1 for the abbreviations of plastic polymers.

Microplastics in the faecal samples and procedural blank samples were identified using Raman spectroscopy. Raman spectroscopic measurements were performed with a Renishaw inVia confocal Raman microscope coupled with a near-infrared 785 nm laser source at 300 mW output power (Wotton-under Edge, UK) and a Leica 10× objective lens (Wetzlar, Germany). Raman spectra of all particles in the area coated with microplastics (8 mm in diameter) on each stainless-steel sieve were acquired using an automated mapping approach at a spatial resolution of 28.4 µm for 5 s per measurement with 10% laser power in the wavenumber range of 676–1767 cm^−1^. Calibration was performed using the vibrational band at 520 cm^−1^ of a silicon reference. Baseline correction, smoothing and cosmic ray removal of the acquired spectra were performed with the Renishaw WiRE 5.2 software. A two-dimensional array of Raman spectra was generated for the area of interest, from which the polymer types of microplastics were identified with the corresponding Raman peaks and were colour-coded using the Renishaw Polymeric Materials Database (Figure 1 and Figure 2) [5]. As microplastics are typically detected as fragments and fibres, the sizes of fragments and fibres were defined as the Feret diameter and length along the central axis, respectively. Size measurements were performed on stereomicrographs of microplastics using the software ImageJ 1.52q (National Institutes of Health, Bethesda, MD, USA).

## 3. Results and Discussion

**Figure 3:**
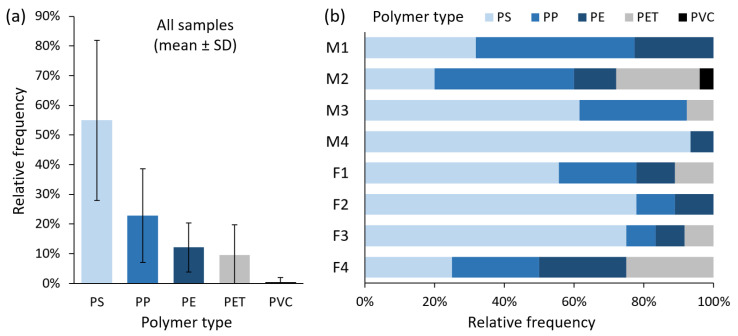
Polymer types of microplastics in (**a**) all faecal samples (*n* = 8) and (**b**) each of the samples, from four men (M1–4) and four women (F1–4), Hong Kong residents. The most abundant type was PS, followed by PP, PE and PET, while PVC was the least common among the samples. Please refer to Figure 1 for the abbreviations of plastic polymers.

**Figure 4:**
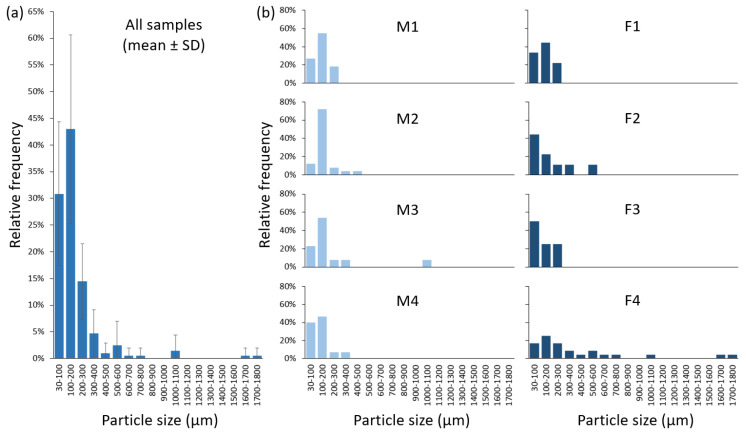
Particle size distribution of microplastics in (**a**) all faecal samples (*n* = 8) and (**b**) each of the samples from four men (M1–4) and four women (F1–4). Particle sizes ranged between 30 and 1800 µm, while microplastics of 30–300 µm accounted for more than 88% of the total number.

Previous investigations on microplastics in human faeces primarily employed the oxidising agent H_2_O_2_, solely or with other chemicals in the biomass digestion step [6,8,10]. Other adopted approaches included digestion in KOH [9] and nitric acid [7] to extract microplastics. The performance of different digestion methods on biological samples has been reviewed (e.g., [15,16]), among which the use of alkalis was the most common, while the use of acids could decompose certain plastic types and thus should be avoided if practicable. Based on these findings, our previous work developed an improved digestion method to extract microplastics from biological matrices using KOH, H_2_O_2_ and EDTA [13]. In the present study, we further modified this method by adding AMIM-Cl to facilitate digestion of cellulose fibres in human faecal samples, where a total of 129 microplastic particles were identified, with the sizes ranging from 40.2 to 4812.9 µm. After correction from the procedural blank samples, 20.4–138.9 particles per g faeces were detected in all faecal samples. Five polymer types were identified, with polystyrene (PS) being the most abundant (55.0% ± 27.0% relative frequency), followed by polypropylene (PP; 22.9% ± 15.8%), polyethylene (PE; 12.1% ± 8.2%), polyethylene terephthalate (PET; 9.5% ± 10.2%), and polyvinyl chloride (PVC; 0.5% ± 1.4%) (Figure 3). The Raman spectra of these polymers are provided in Figure 2. All detected particles of PS, PP, PE and PVC were in the shape of fragments, whereas 66.7% of PET were fibres. The majority of microplastics were 30–100 µm (30.9% ± 13.5%), 100–200 µm (43.0% ± 17.7%) and 200–300 µm in size (14.4% ± 7.1%), with the remaining between 300–400 µm (4.7% ± 4.4%) and 400–1800 µm (7.0% ± 11.4%; Figure 4).

Several recent surveys of microplastics in human faeces reported an average of nine particles per g faeces, which was considerably lower than the average 50 particles per g faeces determined in the present work (Table 1). It should be noted that we set the lowest end of particle size range to be 30 µm, but other studies of microplastics in human faeces adopted different lowest ends at 20 µm [10] and 50 µm [6]. Nevertheless, even if we only counted microplastics > 50 µm (117 out of 129 particles; 90.7%), our results still presented a higher number of microplastics per g faeces than these studies. While the differences could be attributable to methodological variations such as particle size cut-offs and the potential underestimation by Fourier-transform infrared spectroscopy compared with Raman spectroscopy [17,18], our findings nevertheless allude to a higher prevalence of microplastics in the human gastrointestinal tract than is currently appreciated. The same four major types of microplastics were identified among the present and previous surveys (PS, PP, PE and PET; Table 1), but in different proportions where PS was the most abundant in this Hong Kong cohort compared with PP being the most common in other Asian and European individuals [6,7,10]. Such variability in composition might be specific to local environments and lifestyles such as the widespread use of PS takeaway containers in Hong Kong.

## 4. Conclusions

Our findings from faecal samples reveal the potentially high ingestion rates of microplastics by Hong Kong residents, which could be five times higher than those in other places in Asia and Europe. However, larger sample sizes are required in future studies to confirm these findings. Other potential research topics are to establish how the quantities and types of microplastics in the gastrointestinal tract would change in response to dietary or environmental exposure, their perturbations to gut microbiota, and more importantly, whether these microplastics could adversely impact human health. 

## Figures and Tables

**Table 1:** Selected studies of microplastics in faeces of healthy human cohorts in Asia and Europe.

Sampling Region	China (Hong Kong)	China (Beijing)	Indonesia (a Rural Village of Pacet)	Japan (Tokyo) and Europe (7 Cities ^1^)
Sample size and gender	4 men and 4 women	24 men	5 men and 6 women	3 men and 5 women
Years of age	30–65	18–25	20–50	33–65
Prevalence of MP ^2^	100%	96%	64%	100%
Quantity of MP, range	20.4–138.9 particles g^−1^	1.0–36.0 particles g^−1^	6.9–16.5 µg g^−1^	0.8–41.6 particles g^−1^
Quantity of MP, mean ± SD	50.3 ± 39.0 particles g^−1^	8.9 ± 8.5 particles g^−1^	12.2 ± 4.1 µg g^−1^	9.3 ± 14.8 particles g^−1^
Quantity of MP, median	36.4 particles g^−1^	6.5 particles g^−1^	12.4 µg g^−1^	2.0 particles g^−1^
Detected size range of MP	30–1800 µm	20–800 µm	Not reported	50–500 µm
Major polymers of MP	PS > PP > PE > PET ^2^	PP > PET > PS > PE	PP > PE > PS > PET	PP > PET > PS > PE
Major shapes of MP	Fragment > fibre	Not reported	Not reported	Fragment and film > sphere and fibre
Spectroscopic approach	Raman	FTIR ^2^	Raman	FTIR
Reference	Present study	[10]	[7]	[6]

^1^ United Kingdom (Birmingham), The Netherlands (Groningen), Italy (Sassari), Austria (Vienna), Poland (Toruń), Finland (Enontekiö) and Russia (Krasnoyarsk); ^2^ Abbreviations: microplastics (MP), polystyrene (PS), polypropylene (PP), polyethylene (PE), polyethylene terephthalate (PET) and Fourier-transform infrared (FTIR).
